# Nurses' adherence to the Kangaroo Care Method: support for nursing care management^1^

**DOI:** 10.1590/0104-1169.0339.2579

**Published:** 2015-07-03

**Authors:** Laura Johanson da Silva, Josete Luzia Leite, Carmen Gracinda Silvan Scochi, Leila Rangel da Silva, Thiago Privado da Silva

**Affiliations:** 2 PhD, Assistant Professor, Escola de Enfermagem Alfredo Pinto, Universidade Federal do Estado do Rio de Janeiro, RJ, Brazil; 3 PhD, Professor, Escola de Enfermagem Anna Nery, Universidade Federal do Rio de Janeiro, RJ, Brazil; 4 PhD, Full Professor, Escola de Enfermagem de Ribeirão Preto, Universidade de São Paulo, PAHO/WHO Collaborating Centre for Nursing Research Development, Ribeirão Preto, SP, Brazil; 5 Post-doctoral fellow, Escola de Enfermagem, Universidade Federal da Bahia, Salvador, BA, Brazil. Professor, Escola de Enfermagem Alfredo Pinto, Universidade Federal do Estado do Rio de Janeiro, RJ, Brazil; 6 Doctoral student, Escola de Enfermagem Anna Nery, Universidade Federal do Rio de Janeiro, RJ, Brazil

**Keywords:** Neonatal Nursing, Intensive Care Units, Neonatal, Kangaroo-Mother Care Method, Personnel Administration, Hospital

## Abstract

**OBJECTIVE:**

construct an explanatory theoretical model about nurses' adherence to the Kangaroo Care Method at the Neonatal Intensive Care Unit, based on the meanings and interactions for care management.

**METHOD:**

qualitative research, based on the reference framework of the Grounded Theory. Eight nurses were interviewed at a Neonatal Intensive Care Unit in the city of Rio de Janeiro. The comparative analysis of the data comprised the phases of open, axial and selective coding. A theoretical conditional-causal model was constructed.

**RESULTS:**

four main categories emerged that composed the analytic paradigm: Giving one's best to the Kangaroo Method; Working with the complexity of the Kangaroo Method; Finding (de)motivation to apply the Kangaroo Method; and Facing the challenges for the adherence to and application of the Kangaroo Method.

**CONCLUSIONS:**

the central phenomenon revealed that each nurse and team professional has a role of multiplying values and practices that may or may not be constructive, potentially influencing the (dis)continuity of the Kangaroo Method at the Neonatal Intensive Care Unit. The findings can be used to outline management strategies that go beyond the courses and training and guarantee the strengthening of the care model.

## Introduction

Professional adherence does not involve watertight, preprogrammed behaviors to comply with normative requirements, but is directly related to the professional's insertion in the world, in society, in work and potential to transform him/herself, the practices and the context. The need to investigate this phenomenon derives from the immense challenge to make the recommended conducts, which are considered as scientifically protective and beneficial (to the patient and/or professional), permeate the practice as intensely as they are theoretically accepted.

In the context of the Neonatal Intensive Care Unit (NICU), nursing care management should not only meet the technological and infrastructural advances, but also the integrality of care, as a guiding axis of the work processes. Within this logic, the workers, managers and infants/relatives should be considered as protagonists in the production of health^1^.

What the search for quality in neonatal care is concerned, the Kangaroo Method is highlighted, which involves new forms of practicing and thinking care at the NICU, demanding a transformation of the care model in place and of the professionals' conceptions. The adoption of the Kangaroo Method essentially aims to change attitudes towards care and handling the baby and towards the family's participation. This objective, in turn, encompasses some aspects of professional knowledge/actions, like the communicative and creative approach, the opening of spaces and the relation established for care^2^.

The nurse plays a fundamental role in the management of welcoming care, comfort, stimulation and environmental interventions, so as to promote skin-to-skin contact, the infant's development and the strengthening of affective bonds in the family^3^.

Despite the occurrence of important changes in the political and care spheres, in search of quality in neonatal care and care management, the practices are frequently disjointed from the perspective of humanization and integrality. As a proposal for change, the Kangaroo Method has faced challenges, especially to achieve the compliance of professionals who are sufficiently sensitized for this new view and drive the transformation process of neonatal care. This study concerns the first phase of the Kangaroo Method, specifically when the infant is at the neonatal service. The results are expected to contribute to the improvement and implementation of this care strategy, in line with the humanization and integrality policy of care delivery to premature infants with low birth weight, their parents and family.

The objective in this study is to construct an explanatory theoretical model of NICU nurses' compliance with the Kangaroo Method, based on the meanings and interactions for care management.

## Method

This study is part of the interpretative epistemological branch of qualitative research. The theoretical framework was Symbolic Interactionism, which permitted exploring the nurses' compliance with the Kangaroo Method as a phenomenon related to the meanings their interactive activities produce in care. In function of the symbol, the human being does not respond passively to the reality, but actively and creatively, always recreating the world of action^4^.

Grounded Theory was the methodological framework selected because of its high level of systemization in the interpretation of the data, which aims to produce constructs or theoretical models, explaining the action in the social context. Due to the intensity of its analytic rigor, it demands theoretical sensitivity, creative attitude and determination from the researcher^5^^-^ 6.

The field work took place in the second semester of 2011 and the first semester of 2012 and was assessed by researchers trained in the method. The scenario was an NICU of a public university hospital in the city of Rio de Janeiro (RJ), which is a referral institution for high-risk pregnancy, especially regarding fetal risk. The three phases of the Kangaroo Method has been implemented since the year 2000. Eight nurses participated in the study, who complied with the following inclusion criteria: having more than one year of experience in the neonatal intensive care area; having at least six months of experience at the institution's NICU and being familiar with the first phase of the Kangaroo Method. Professor on medical leave during the data collection did not participate in the research, which was the only exclusion criterion.

The data collection technique used was the in-depth interview, with voice recording and validation by the participants. The instrument was submitted to a pilot test, including a short characterization of the participant and guiding questions: "Talk about the meanings of the Kangaroo Method for you in your work at the NICU and how you develop it with your team". The participants' statements were identified by codes, whose letters refer to the pseudonyms chosen and the numbers to the sample group they were part of.

The interviews were closed off based on theoretical saturation, which was made possible by the simultaneous data collection and comparative analysis. The representativeness of the subjects and the information quality were used as criteria for analytic density in the theoretical sampling process^7^.

The data were submitted to three coding phases: open, axial and selective. The obtained codes were submitted to comparisons and inductive grouping in subcategories and categories. Through the integration and refining of the categories, a theoretical conditional-consequential model could be developed. The writing of memoranda and the construction of diagrams were two analytic techniques used in all coding phases and permitted maintaining the roots in the data^5^.

The theoretical model obtained was validated by 17 professionals from different categories and institutions. For the purpose of representativeness and range, three Brazilian tutors of the Kangaroo Method participated in this group, one state tutor (affiliated with the research institution) and six nurse managers of public neonatal services in the city of Rio de Janeiro. This step granted credibility to the study.

All ethical aspects were complied with in accordance with Resolutions 196/96 and 466/12. Approval for the project was received from the Research Ethics Committee of the Teaching Maternity at Universidade Federal do Estado do Rio de Janeiro, under opinion 20/2011. The participants signed the Free and Informed Consent Form, guaranteeing the freedom to participate and preserving their identity.

## Results

All^8^ participants were female, with ages ranging between 20 and 45 years and a mean age of 39 years. These nurses' time since graduation ranged between 4 and 19 years, with a mean six years of experience in the maternal-infant area. Most nurses^7^ indicated a specialization degree in areas related to their activities and all of them indicated theoretical and/or practical training in the Kangaroo Method. As regards the scale and function, four participants worked during the day (first sampling group) in supervision and management and four worked as duty nurses (second sampling group), executing care and team leadership activities.

Four main categories emerged from the coding process which integrate the main concepts of this research and contain the relations among the symbols, actions, social interactions and meanings that emerged in daily work at the NICU.

### Giving One's Best to the Kangaroo Method

The symbolic action of giving one's best (in vivo code) to the Kangaroo Method implies interactional communication with the others about the philosophy defended and the ways of delivering care to low-weight infants and their families at the NICU.

The compliance attitudes the nurses mentioned were: having a new perspective, believing, participating and interacting in team. The internal attitude of believing in the Kangaroo Method and valuing it derives from experience, that is, from the possibility of trying it out in care practice. Thus, the professionals give credit to what they experience and not necessarily to what is simply presented to them in courses and training. *For me, believing is being sure that the Method works. We believe in what we have used, tried out and proved *(Nurse D2)*.*

The implementation is based on the combination of various professionals' efforts who believe in the Kangaroo Method philosophy (Love, Warmth and Breast Milk) and work to overcome the challenges, engage responsibly and make the work in the first phase happen with optimism and determination.

With a view to the team's compliance, the nurses highlight the importance of contagion (in vivo code), when one or more experienced professionals engaged in the application are able to disseminate the philosophy and practices among the colleagues, whom they interact with in daily work at the NICU. *So when I talk about giving one's best that's it, it means believing and going for it. Believing and aiming for that by all means and contaminating other people. Contaminating to do what is best really, where the people will also want to be part of this team, do their best *(Nurse C1)*.*

### Working with the complexity of the Kangaroo Method

The characterization of the complexity involved in the subjects' discourse refers to the multiplicity and dynamic behavior of the care and management demands in this care model at the NICU. It involves broad and interlinked issues, such as care, citizenship and affection, which occupy a singular place in the concreteness of care, as each person has his/her history.

These issues drove the nurses to conceive new meanings for work at the NICU. *So I think that humanizes our work, it's not just noise, aspiration, machine. It mitigates, as if everything were becoming warmer. [...] All bad things that may be happening in terms of work, of difficulty for me, watching and perceiving how that is working it, mother and child in that position (skin to skin), at that moment, it makes me feel relieved [...]* (Nurse J2).

The nurses presented a risk conception that goes beyond the biological aspects. In their practices, they demonstrated concern with the vulnerabilities of the premature infant's family and with care that positively influences the health and quality of life after hospital discharge, especially in terms of childhood growth and development.

To practice the Kangaroo position, the nurses mention dealing with clinical criteria and with the parents' subjective aspects. Welcoming, communication, promotion of affective bonding, stimulation of maternal participation and safety, minimization of the infant and the family's suffering/stress were the main areas highlighted in the nurses' activities in the Kangaroo Method. *As soon as she starts to do it (Kangaroo position), the mother feels more confident that that baby, despite being low-weight, that she can keep him outside the incubator for some hours. [...] So after they start doing the Kangaroo position, I see that's a start, it's a new course *(Nurse S1).

### Finding (de)motivation to apply the Kangaroo Method

One important aspect linked to the compliance process is the professionals' motivation to apply the Kangaroo Method. The discourse appointed the need to value the nursing professionals' individual preferences and aptitudes for work at the NICU.

The attraction and affinity with the type of work was a fundamental aspect in the motivation process and in the decision to develop the Kangaroo Method. The nurses acknowledged that this attraction towards humanizing practices is linked to the life philosophy, knowledge baggage and emotional response of the professional to the stress deriving from work. *It depends on the person's spirit, I'd say that from the philosophy too and from that influence during education, during training. I'd say the philosophy of living, of relating, of humanization. [...] Because there are people who are able to get both sides, they're intensive care nurses and are also able to be patient to do it (Kangaroo Method), but you observe that there are people who do it, with kindness and everything, but say: Ai! I wasn't born for this, I prefer an intubated child in severe conditions, because I go there and do it *(Nurse R2).

The main sources of motivation to develop the Kangaroo Method were the verification of the quality and humanization of care, the maternal acknowledgement and the family members' affect, the good care results verified upon discharge from the NICU and the professional's satisfaction when observing other colleagues' engagement, affect and pleasure in the application of the Kangaroo Method. *What motivates me is to see a healthy child in the future, knowing that I am contributing to that child's future, a healthy future* (Nurse D2).

The main sources of demotivation to develop the Kangaroo Method were the discording values in intensive care, the distorted conceptions about the Kangaroo Method among the heads and colleagues, the mismatch between the work and the objectives and the devaluation of the Kangaroo Method by professionals who do not like and do not believe in the model. *What discourages me is to see that it does not receive due importance (cry). [...] You deliver care with so much kindness! We know it's important, why don't people consider it as important as it actually is?*(Nurse A1).

### Facing challenges for compliance and application of the Kangaroo Method

Daily nursing care at an NICU is very complex and entails countless challenges that demand a wide range of knowledge, skills and attitudes from the professionals. The lack of time was highlighted as a limiting factor for the practice of the Kangaroo Method due to the intense dynamic/routine, work burden and lack of human resources, leading to the professionals' limited availability to be present and dedicate themselves to time-consuming care (listening and support). *So, welcoming demands time, availability and inside the first (first step at NICU) you don't have, sometimes due to human resources, no matter what, you don't have that time available to sit down with that mother, to put the infant at the breast, to wait until the infant wants to latch on, when he sleeps and wakes up (*Nurse C1).

Other difficulties mentioned were the agitated and noisy environment of the NICU, the lack of technical confidence and mismatch among the professionals (nursing and other categories) to practice the Kangaroo Method, the extreme valuation of the biological view, the existence of attitudes of indifference, disinterest and lack of commitment by some professionals, the resistances and limitations some physicians impose and the difficulty to work in teams. *The Kangaroo Method came to help us and develop that practice better for the infants, but people don't want it. [...] Some professionals do it and really feel gratified, even professionally, as a person. But others unfortunately really don't care (*Nurse E2).

The Kangaroo Method practices at the NICU are marked by discontinuity, among the professionals and shifts, which is associated with a lack of charges, supervision and standardization. The nurses mentioned management difficulties and highlighted the need for strategies with a view to safer leadership, greater team motivation and coping with conflicts. *I think the leadership would need to happen in that sense of stimulating people to put the Method in practice So, you doing it, saying: look, we're gonna do it now with that mother and so..., really insist* (Nurse AM1).

### The theoretical model

In the Grounded Theory, the central phenomenon is identified based on the search for connections between the categories and the theme of the type of relationship that joins them. It translates the most relevant and often most frequent theme in the occurrence of the data^8^. The comparative interpretation of the data evidenced the following central phenomenon, as a multiplier of values and practices for the (dis)continuity of the Kangaroo Method at the NICU. The idea of multiplier adopted in this study is that of a professional who introduces and disseminates new forms of seeing and coping with the problems in his/her group and greater opening towards changes in practice. Thus, multipliers have the ability to transform, as they influence the reorganization of the social reality they are part of, constructing a shared view of reality with the other stakeholders^9^.

The central phenomenon highlights two important elements that, in combination, sustain the transformation of daily care, valuation and continuity. This gives rise to two important possibilities: multipliers who value the model and disseminate positive value, contributing to the continuity of the Kangaroo Method in their practice; and, at the other end, multipliers who devalue the model and disseminate negative values that cause demotivation and favor the discontinuity of the care model. Thus, compliance finds its truest meaning in the interaction with other professionals and involves issues of value, profile, dedication, knowledge, relationship and commitment.

**Figure 1.**
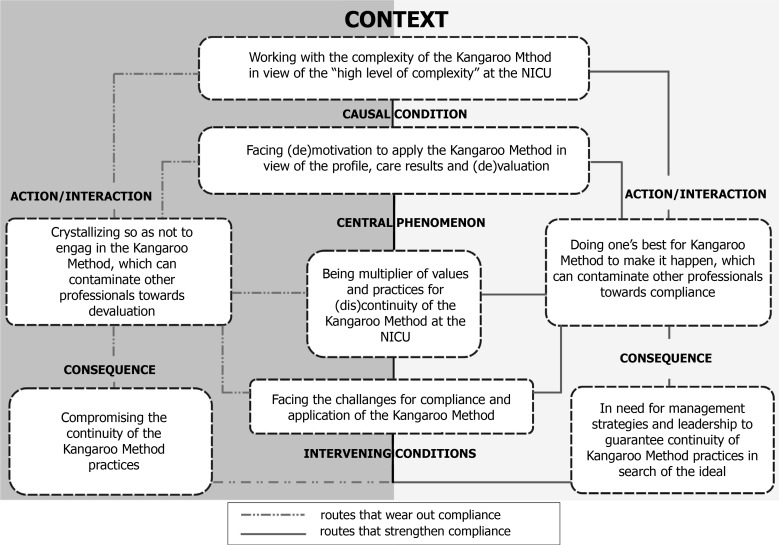
Theoretical model of compliance with the Kangaroo Method

The link among the categories in this research was based on the Conditional/Consequential Paradigm or Model, which offers an organization scheme to join and order the data, so as to integrate structure and process^5^. The diagram (Figure 1) schematically represents the (converging or diverging) interactions that strengthen or wear out the dynamic process of professional compliance with the Kangaroo Method.

## Discussion

From the perspective of symbolic interactionism, not only the individual and the influence of the social structure or his/her personality on individual behavior is focused on, but the nature of the interactions and the dynamic social activities between the people in a society^4^. Thus, the adherence to the Kangaroo Method can be conceived as a result of the interactions among the professionals at the NICU, being therefore dynamic, social and intersubjective.

In the application context of the humanizing practices, there is a distance among prescriptive, institutionally established and truly executed work. This contradiction happens in the face of a hierarchical and generally centralizing work structure, where the professionals are more valued for their mastery of the technological environment, for the execution of standardized and routine tasks than for relational actions^10^.

Sporadic and inconsistent actions, deficiencies in the professionals' education and experience to take care of families, organizational issues, such as the availability of resources and staff, the culture itself and commitment to work represent difficulties for the development of family-centered models. The inclusion of the family in the intensive care environment implies the redefinition of the professionals' functions and the adoption of humanization as a central view in professional education and practice^11^^-^ 13.

The predominant values in education and practice derive from the hegemonic medical model and influence both the public work process and each worker's private practice. Health work cannot be fully controlled, as it is put in practice continuously through the relation between people, being therefore subject to the way the professionals develop their practice. Hence, within their autonomous space, the professionals act with intentions that are based on their values and conceptions^14^.

The way each person feels, thinks and acts is permeated by that person's intentions and interests. This private universe composes the organization's psychosocial dimension, which is dynamic because people continuously change when interacting, also modifying the reality^15^. Therefore, it is important to establish a collaborative procedure change, in which the leaders and their subordinates jointly constitute a new individual and collective culture, actively participating in the process and acknowledging the presence of tensions. One of these tensions is the dynamics of work at the NICU as a source of pleasure and burnout for professionals^16^.

Studies undertaken at neonatal services have revealed professionals' lag with regard to the high work demand they are submitted to each day, the lack of material, limited qualification of technical staff, overcrowding, inappropriate facilities, lack of continuing education, flaws in team communication and absence of a care protocol^17^^-^ 18.

In view of this reality, the central role of management in nursing care as a process should be acknowledged, with great potential to lever changes. Improvement actions in care fully depend on the nurses' better performance in service and team management, adopting postures of leadership and commitment to the profession and to the work practice^19^.

Management is a powerful instrument to trigger a critical reflection process on daily practice in the teams, favoring the professionals' compliance and commitment to a better care production process, beyond fragmented tasks and procedures^20^.

With a view to the dissemination, implementation or strengthening of the Kangaroo Method, managers and leaders need to understand the importance of (individual and organizational) core competences that will permit advances in the achievement of targets and outcomes. Management practice needs to be directly related to staff development in the job world, promoting an organizational culture that values qualities beyond the technical competences. This is a constructive and positive culture, based on conquests and the encouragement of humanism, with a special place for creativity, emotion and relationship^21^^-^ 22.

For the changes in nursing practice to happen, a strategy needs to be established for the preparation of leaderships who assume innovative ideas, enhance creative environments and break with the barriers that impede organizational changes^23^.

Depending on his/her profile, each professional can have experiences in the same context that link or distance him/her from the care model. The (re)construction of the meanings for work in the Kangaroo Method derive from social interaction. Amidst these multiple experiences, the process of being a multiplier emerges, whose active participation is essential for the changes, according to the results of this research.

Through a more participatory management practice, the nurses can intensify the work of the teams in the Kangaroo Method, granting them opportunities to experience challenges that make the work more attractive, with a view to a more qualitative, comprehensive and humanized care.

## Conclusion

The understanding that emerged from this study is that the professionals' compliance process is dynamic and consists of choices, interactions and meanings, related to their engagement in the Kangaroo Method. The intensity of this link can vary depending on the professional's subjectivity and profile and on the objectivity (structure and process) of the work, taking the form of values, attitudes and actions in the symbolic space of the interactions with the infant, the family and other professionals.

In that context, which is individual and collective at the same time, the adherence to the care model is a phenomenon that is particularly put in practice in the field of the influence on other professionals. That is why the role of multipliers was highlighted in the testimonies of the nurses who are concerned with conquering more professionals to transform the practices and strengthen the Kangaroo Method.

Thus, a theoretical model was proposed in which the role of multiplier is presented as the baseline process for this phenomenon, assumed based on the nurse's motivation to think and act with regard to the Kangaroo Method. The nurse and her team's link with the care model receives influence from the confrontation, at the symbolic level, between the ideal, established by policies, and the reality, circumscribed to the concrete possibilities of practice.

In view of the difficulties, the nurses highlighted the need for a changed perspective and attitudes in care, in view of the significant resistance to the Kangaroo Care practices that still exists at the NICU. Far beyond the lack of knowledge about the model and its theoretical-philosophical background, this resistance is linked to the professional-centered perspective, according to which care is delivered without first considering the infant and the family's individual needs. In addition, the intensive care routine imposes a rhythm of chronologically defined procedures and tasks to which many professionals respond with automated care.

In the symbolic interaction among the nursing team members, some value the Kangaroo Method and insist on its application, despite the difficulties. Others devalue and compromise the continuity of these care practices at the NICU. In general, the latter are crystallized in the practices of the model in force and are resistant to attempts to change. Both positions are characterized by these multipliers' (whether favorable or unfavorable to the model) strong influence on the meanings the other team members attribute to the Kangaroo Method, involving values, attitudes and practices. This conflicting context deriving from divided opinions and practices in the teams gives rise to the urgent need for management strategies that are specifically focused on strengthening the Kangaroo Method at the NICU and on the professionals' compliance. These strategies will guarantee the continuity of these care practices, so that the operation of the Method is not limited to some few professionals' choice, but is part of a comprehensive care framework involving the teams.

Further research is recommended to understand the relation between the profile of the professionals who work in neonatal intensive care and competency development for humanization practices, as well as the design of management and training strategies that strengthen the Kangaroo Method.
